# Breaking the Barrier in Conversion of Methane to Ethanol: A Molecular Junction Photocatalyst

**DOI:** 10.34133/research.0890

**Published:** 2025-09-16

**Authors:** Lingyi Li, Yingkui Yang, Xueiqn Liu

**Affiliations:** State Key Laboratory of New Textile Materials and Advanced Processing, Wuhan Textile University, Wuhan 430200, Hubei, China.

Photocatalytic methane oxidation into value-added products represents a promising pathway for the sustainable chemical production. However, achieving high reaction selectivity with efficient solar energy utilization continues to be a major obstacle in photocatalytic process. In a study led by Tang and coworkers, an intramolecular heterojunction photocatalyst with alternating benzene and triazine motifs was introduced to convert methane into ethanol with high selectivity and conversion efficiency.

Methane (CH_4_), the primary constituent of natural gas, is a potent and widely prevalent greenhouse gas with a global warming potential 25 times greater than carbon dioxide (CO_2_). The conversion of CH_4_ into high value-added liquid fuels (e.g., CH_3_OH and C_2_H_5_OH), which are transportable and storable, holds great promise for the efficient use of fossil fuel feedstocks and the reduction of greenhouse gas emissions [1]. Nonetheless, traditional thermal catalytic approaches require harsh conditions, such as high reaction temperatures and pressures. Solar-driven photocatalysis emerges as a highly attractive route for mild-condition CH_4_ activation, which has attracted increasing attention [2–4]. Various photocatalysts, especially for the polymeric carbon nitride, including copper-modified carbon nitride, nitrogen-rich carbon nitride, and phosphorus-doped carbon nitride, have been explored for their ability to drive the photocatalytic conversion of CH_4_ into multi-carbon products. However, the efficient C_2_ product formation of photocatalytic CH_4_ oxidation reaction is still limited by (a) the large energy barrier for the initial C–H activation, (b) the overoxidation to CO₂, and (c) the poor C_2_ product selectivity due to the lack of binding sites [5]. The simultaneous achievement of high ethanol selectivity and substantial CH_4_ conversion represents a breakthrough through the innovative photocatalyst design.

**Fig. 1.**
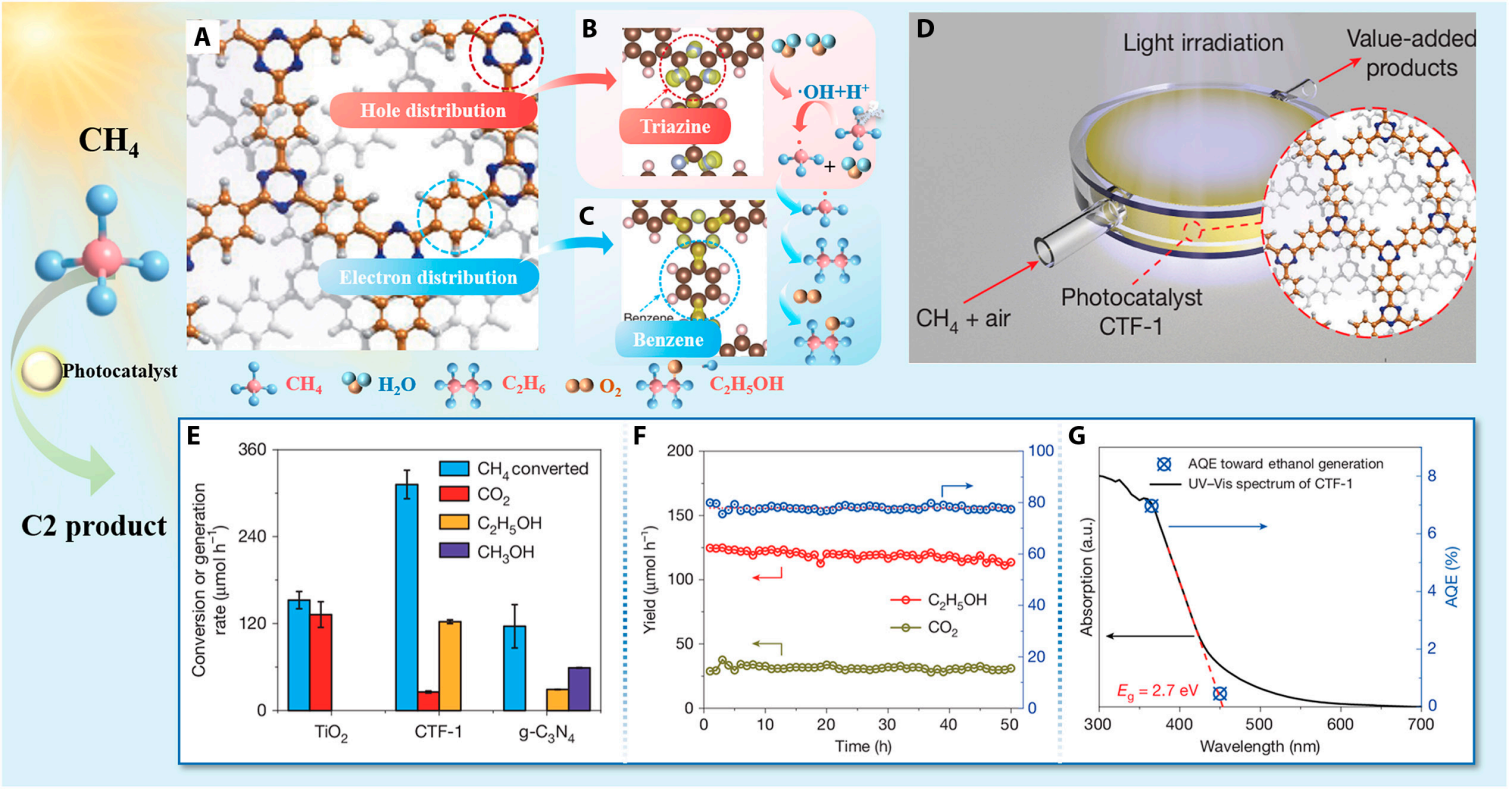
Mechanism diagrams and photocatalytic CH_4_ oxidation performances of CTF-1. (A to C) Schematic diagram of intramolecular junction and spatial distribution of electrons and holes. (D) Schematic diagram of packed-bed photocatalytic reactor. (E) Comparative analysis of photocatalytic CH_4_ oxidation performance: CTF-1, g-C_3_N_4_, and TiO_2_. (F) Stable C_2_H_5_OH production and high selectivity in long-term photocatalytic CH_4_ conversion using CTF-1. (G) AQE of photocatalytic CH_4_ transformation to C_2_H_5_OH by CTF-1. Copyright 2025, Springer Nature.

The recent study by Tang and coworkers [6] designed a covalent triazine-based framework (CTF-1) as an intramolecular heterojunction photocatalyst to achieve selective oxidation of methane to ethanol under mild condition. The innovative architecture of CTF-1 photocatalyst enables precise spatial separation of photo-induced charges during photocatalytic processes through its distinct triazine and benzene motifs (Fig. 1). Near-edge x-ray absorption fine structure spectroscopy provided direct observation of light-induced charge redistribution, with photoelectrons accumulating at benzene motifs and holes localizing on triazine units, respectively. Under light irradiation, the intramolecular junctions were designed to stabilize the holes on the triazine unit and the electrons on the phenyl unit, thus avoiding their rapid combination and improving the photocatalytic performance. The study in situ diffuse reflectance infrared Fourier transform spectroscopy and electron paramagnetic resonance measurements offered key mechanistic validations. The triazine units function dually as (a) the dominant hole accumulation center (Fig. 1) and (b) the active redox site for water dissociation to form •OH radicals. The •OH radicals possess sufficient oxidizing power to cleave the strong C–H bond in CH₄, reducing the activation barrier compared to direct hole-mediated cleavage, thereby facilitating the formation of •CH_3_ radicals. The introduction of benzene provides optimal binding sites for •CH_3_ radicals (−116 kJ mol^−1^ adsorption energy). Newly formed •CH_3_ radicals generated from triazine units migrate to benzene units for C–C coupling to form C_2_H_6_. C_2_H_6_ subsequently reacted with surface-adsorbed O_2_, yielding C_2_H_5_OH and H_2_O (Fig. 1).

Although CTF-1 showed nearly identical adsorption energies for both ethane and ethanol, the O-containing substances adsorbed on the surface facilitated the conversion of ethane to ethanol prior to ethane desorption. Under the competitive reaction, some •CH_3_ radicals may recombine with water-generated •OH radicals to form methanol (−40 kJ mol^−1^) that is strongly adsorbed on the surface of CTF-1 and further oxidized to CO_2_, whereas the weak adsorption of ethanol (−18 kJ mol^−1^) makes it more readily desorbed from CTF-1 and further oxidation is avoided. This explains only trace amounts of ethane on CTF-1 and the high selectivity (78.6%) for ethanol. The design of the CTF-1 molecular junction reduces the recombination of electrons and holes, while the separation of the oxidation and coupling sites is another key design for preventing the peroxidation of the C_2_ production.

The device for the reaction is also important to the catalytic performance. In conventional batch photocatalytic systems, the accumulation of reactive oxygen species (ROS) promotes over-oxidation of primary products (e.g., ethanol), resulting in progressive selectivity loss during methane conversion. To avoid peroxidation of methane, the authors designed the packed-bed photocatalytic reactor operating under ambient temperature and pressure conditions (Fig. 1). The gas-phase system featured a compact polytetrafluoroethylene body with an optical-grade quartz window, creating a 0.6-ml reaction zone densely packed with 1 g of the porous CTF-1 catalyst. A precisely controlled 16:1 methane-to-oxygen mixture (20% CH₄/Ar and humidified synthetic air) flowed through the catalyst bed at 40 ml min^−1^. The packed-bed reactor with continuous-flow configuration overcame classical batch reactor limitations, such as the accumulation of ROS and poor product selectivity, while aligning with net-zero process, no solvent waste, and direct solar energy utilization. However, scaling this reactor design poses significant engineering challenges. The exceptionally high photocatalyst loading combined with suboptimal gas flow rates created significant mass transfer challenges for scale-up, while the exclusive dependence on ultraviolet (UV) irradiation [365-nm light-emitting diodes (LEDs)] introduced critical scalability constraints. Comparatively, alternative designs like fluidized-bed reactors or microstructured systems could enhance contact of gas and photocatalyst and light penetration but exhibited reduced reaction selectivity compared to conventional packed-bed designs. Future reactor designs could integrate packed-bed selectivity with membrane-separated product extraction or graded-porosity architectures to simultaneously optimize mass transfer and photon utilization.

CTF-1 exhibited significantly enhanced photocatalytic activity compared to traditional photocatalysts like TiO_2_ and g-C_3_N_4_. Although both CTF-1 and g-C_3_N_4_ employed water-derived •OH radicals for methane activation, CTF-1 demonstrated superior water adsorption capability compared to g-C_3_N_4_, attributable to its triazine functional motifs. Through its dual hydrogen-bonding interactions, CTF-1 significantly reduced the water dissociation energy barrier from 240 to 129 kJ mol^−1^ while increasing hole transfer rates from 1.7 × 10^8^ to 5.2 × 10^8^ s^−1^. This unique molecular design conferred CTF-1 with superior photocatalytic performance, exhibiting lower water dissociation barrier and higher radical generation efficiency compared to conventional g-C_3_N_4_ systems.

The product selectivity of CTF-1 was compared to that of the traditional photocatalysts anatase TiO_2_ and g-C_3_N_4_ using the packed-bed flow reactor to provide a steady-state reaction environment (Fig. 1). TiO_2_ produced primarily CO_2_, and g-C_3_N_4_ yielded only 20% of CTF-1’s ethanol production rate. By both experiment results and density functional theory calculation, CTF-1 showed lower charge recombination and better water adsorption than did g-C_3_N_4_.

Except for the conversion efficiency, photostability is also an essential factor for evaluating the photocatalytic performance, especially for the future practical applications. Under 365-nm LED irradiation, the amount of methane conversion reached 12,000 μmol during the continuous photocatalytic reaction for 50 h. The comparison of chemical composition and crystalline structure before and after long-term reaction confirmed the excellent stability of the structure of CTF-1. The gas chromatography–flame ionization detection revealed that only CO₂ and ethanol were produced, with a high ethanol selectivity of 75.6% to 80.0% being maintained (Fig. 1). The results clearly indicated the high selectivity and excellent photocatalytic durability of CTF-1. CTF-1 attained 6.9% apparent quantum efficiency (AQE) for ethanol production from methane under 365-nm light (Fig. 1). Notably, when modified with PtO*_x_* co-catalyst, CTF-1 achieved a significantly enhanced AQE of 9.4% at 365 nm, representing a 30-fold improvement over previously reported photocatalytic systems. The PtO*_x_*/CTF-1 system exemplified how atomic-level integration of inorganic co-catalysts with organic frameworks can transcend traditional trade-offs between activity and selectivity in photocatalysis.

Photocatalytic oxidation under ambient conditions presents an energetically favorable pathway for selective methane conversion to value-added products. The work by Tang and coworkers reported an extremely promising methane oxidation to ethanol process over intramolecular junction photocatalyst CTF-1 with efficient charge separation and spatial isolation of redox-active and C–C coupling sites in the molecular level, leading to the highly efficient and selective photocatalytic methane oxidation to ethanol. The current photocatalyst system presents several inherent challenges: (a) narrow solar spectrum utilization, necessitating strategies to enhance visible-light absorption; (b) undesired methanol oxidation pathways that produce CO₂, calling for precise regulation of radical kinetics and tailored active-site design; (c) scalability limitations that hinder practical industrial deployment. To overcome current limitations, next-generation photocatalysts demand precise atomic-level design to achieve dual optimization: reduced activation energies and heightened reaction selectivity. To broaden the spectral response, strategic molecular engineering, such as integrating light-harvesting porphyrin units into covalent organic frameworks, effectively extends photocatalyst activity into the visible range. On top of that, reactor engineering innovations represent a critical frontier in advancing photocatalytic methane conversion, particularly in addressing mass transfer limitations and photon utilization efficiency, such as the design of 3-phase interface systems (gas–solid–liquid) to overcome mass transfer limitations.
